# Supplementary material for: Skyrmions-based logic gates in one single nanotrack completely reconstructed via chirality barrier
Source: Natl Sci Rev. 2022 Feb 18;9(12):nwac021. doi: 10.1093/nsr/nwac021 (PMC9874028; doi:10.1093/nsr/nwac021)
Supplement: nwac021_Supplemental_Files [file nwac021_supplemental_files.zip › Supplementary_Materials_20220105.docx]

**Supplementary Materials for**

**Skyrmions-based logic gates** **in** **one single nanotrack completely reconstructed via chirality barrier**

Dongxing Yu^1^, Hongxin Yang^1,4,*^, Mairbek Chshiev^2,5^, Albert Fert^3^

^1^Quantum Functional Materials Laboratory, Ningbo Institute of Materials Technology and Engineering, Chinese Academy of Sciences, Ningbo 315201, China

^2^Université Grenoble Alpes, CEA, CNRS, Spintec, 38000 Grenoble, France

^3^Unité Mixte de Physique, CNRS, Thales, Université Paris-Sud, Université Paris-Saclay, Palaiseau 91767, France

^4^Center of Materials Science and Optoelectronics Engineering, University of Chinese Academy of Sciences, Beijing 100049, China

^5^Institut Universitaire de France (IUF), 75231, Paris, France

*Email: hongxin.yang@nimte.ac.cn


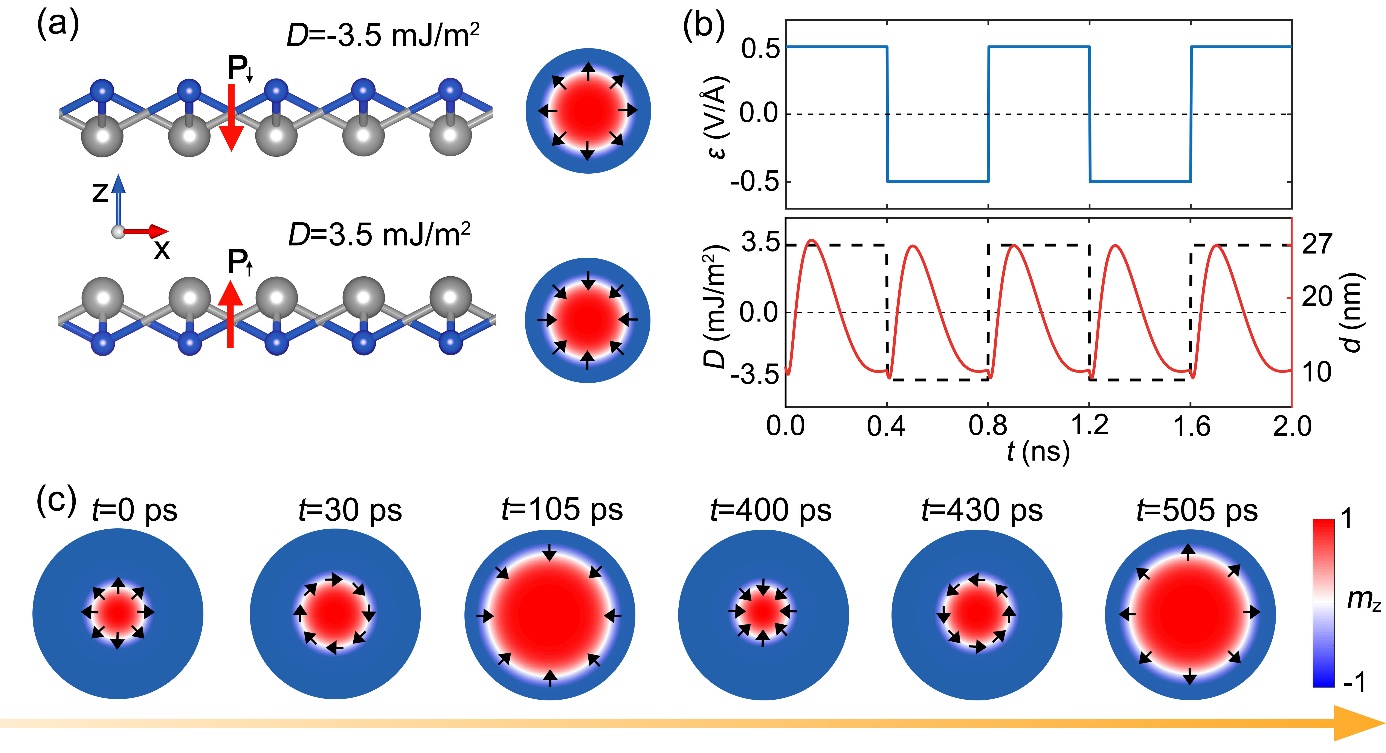


**Figure S1.** Simulated skyrmion chirality switching accompanied by breathing mode. (a) Two-dimensional multiferroics monolayer with controllable fluctuations. The direction of Rashba-type DMI and the chirality of skyrmions can be switched by electric field pulse via electrical polarization **P**. (b) Evolution of the skyrmion diameter *d* and DMI constant *D* (down) driven by electric field $\varepsilon$ (up) as a function of time. Snapshot images in (c) illustrating the skyrmion helicity ($\gamma=0 \to\gamma=-\frac{\pi}{2}\to\gamma=\pi\to\gamma=\frac{\pi}{2}\to\gamma=0$) and size at different stages. With the application of periodic electric field, the subsequent skyrmion dynamic (0.8 ns – 2 ns) also shows a good stability.


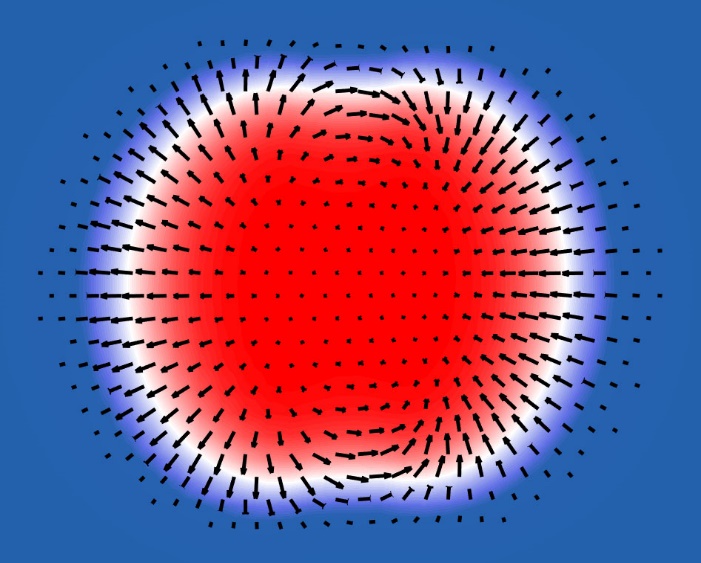


**Figure S2.** Topological non-trivial particle in the OR operation. The magnetization configurations with topological charge Q=1.87, in which two skyrmions with opposite chirality merged together. The merging is the result of the pinning effect of DMI chirality barrier and the mutual attraction of magnetic skyrmions with opposite chirality.


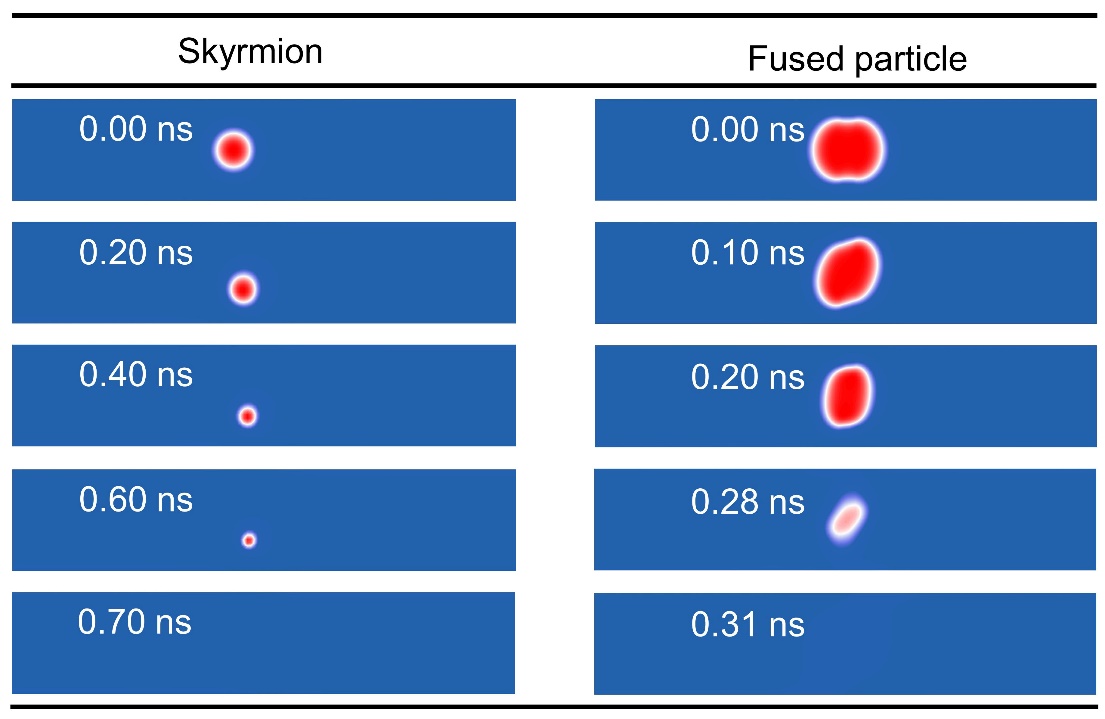


**Figure S3.** Snapshots of simulation at different times for magnetic skyrmion (left panel) and fused non-trivial particle (right panel) erased by a large current density 45 MA/cm^2^.


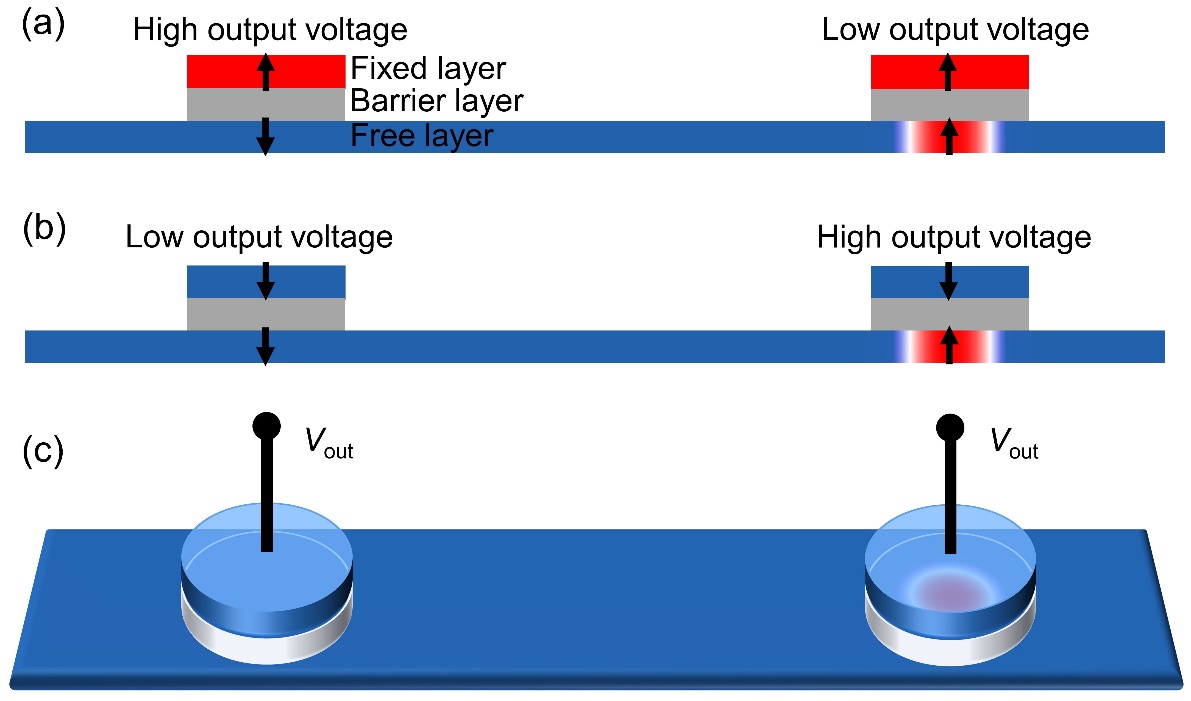


**Figure S4.** Magnetic tunnel junction (output-MTJ) structure. Four different magneto-resistive states and the corresponding output voltages for the fixed layer magnetization pointing (a) up, (b) down. (c) Schematic diagram of detecting the background state (left output-MTJ) and the magnetic skyrmion state (right output-MTJ) in a racetrack.


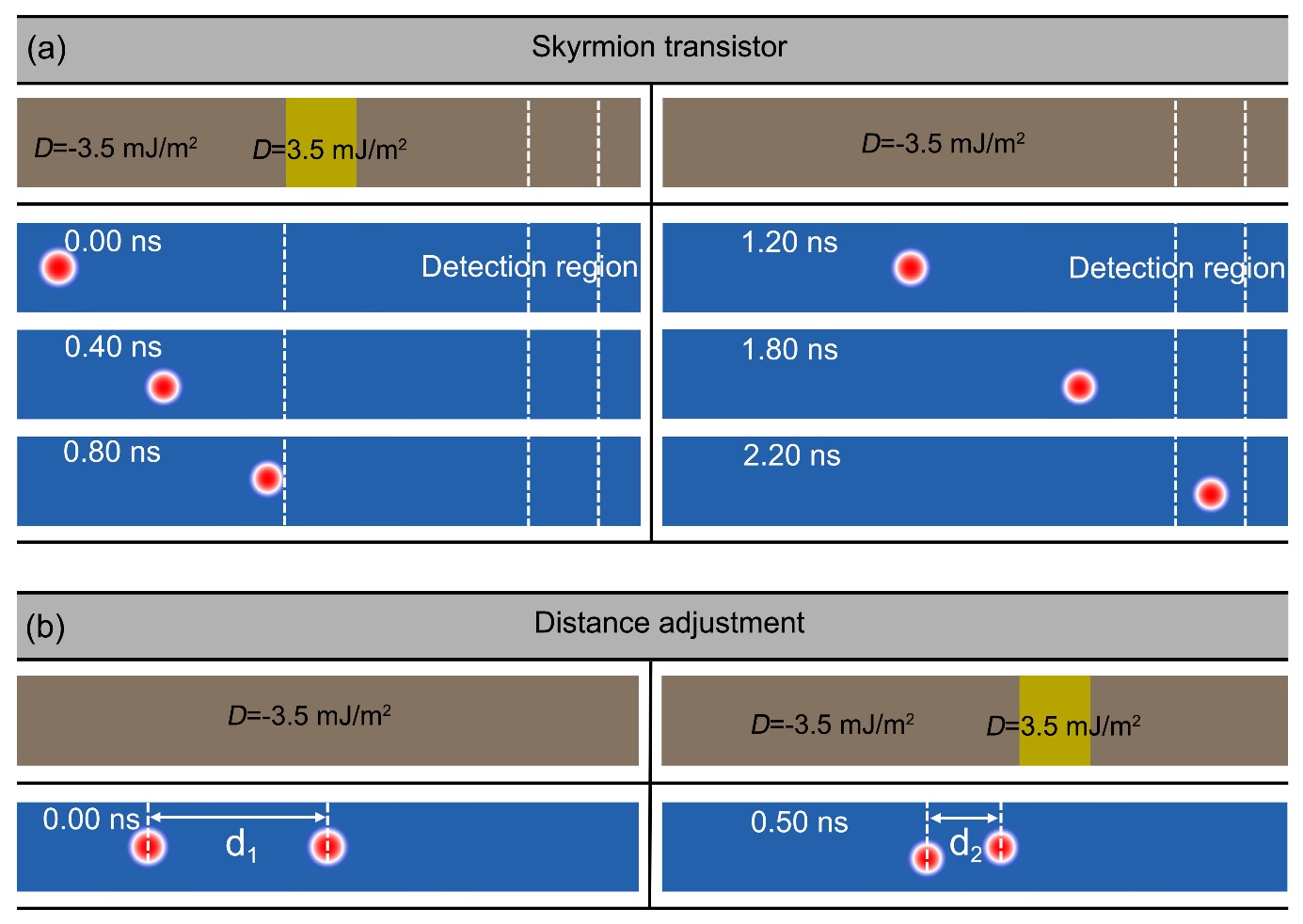


**Figure S5.** Device structures with size 30 nm × 210 nm for skyrmion transistor and skyrmion bit reset. (a) “off” states (left column) and “on” states (right column) of a skyrmion transistor based on the pinning/depinning function of DMI chirality barrier (the brown region in the strip) controlled by electric field pulse. (b) Distance adjustment between skyrmions. The DMI chirality barrier in the right panel pins the right skyrmion. The current drives the left skyrmion to move to the right, shortening the distance between the two skyrmons from d1 to d2.


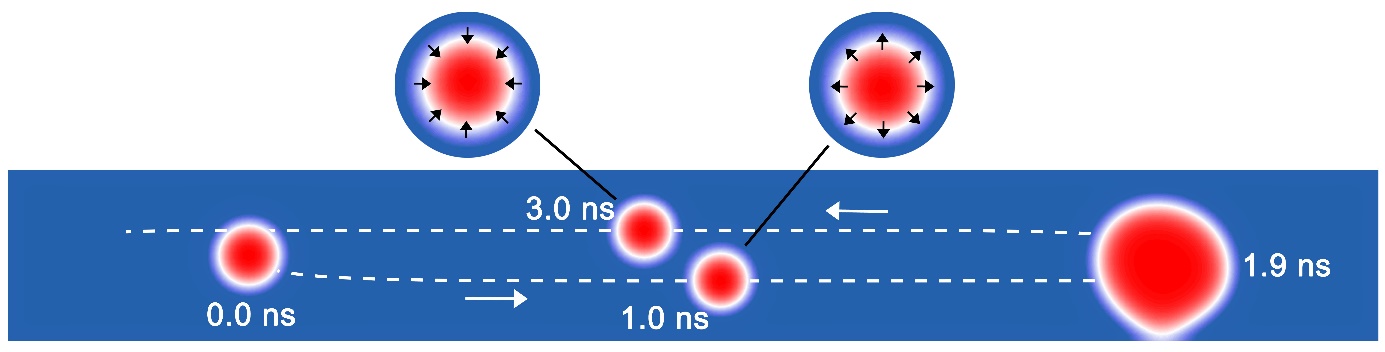


**Figure S6.** Back and forth motion of skyrmion (skyrmion recycling) switched by electric pulse without changing the in-plane current direction.


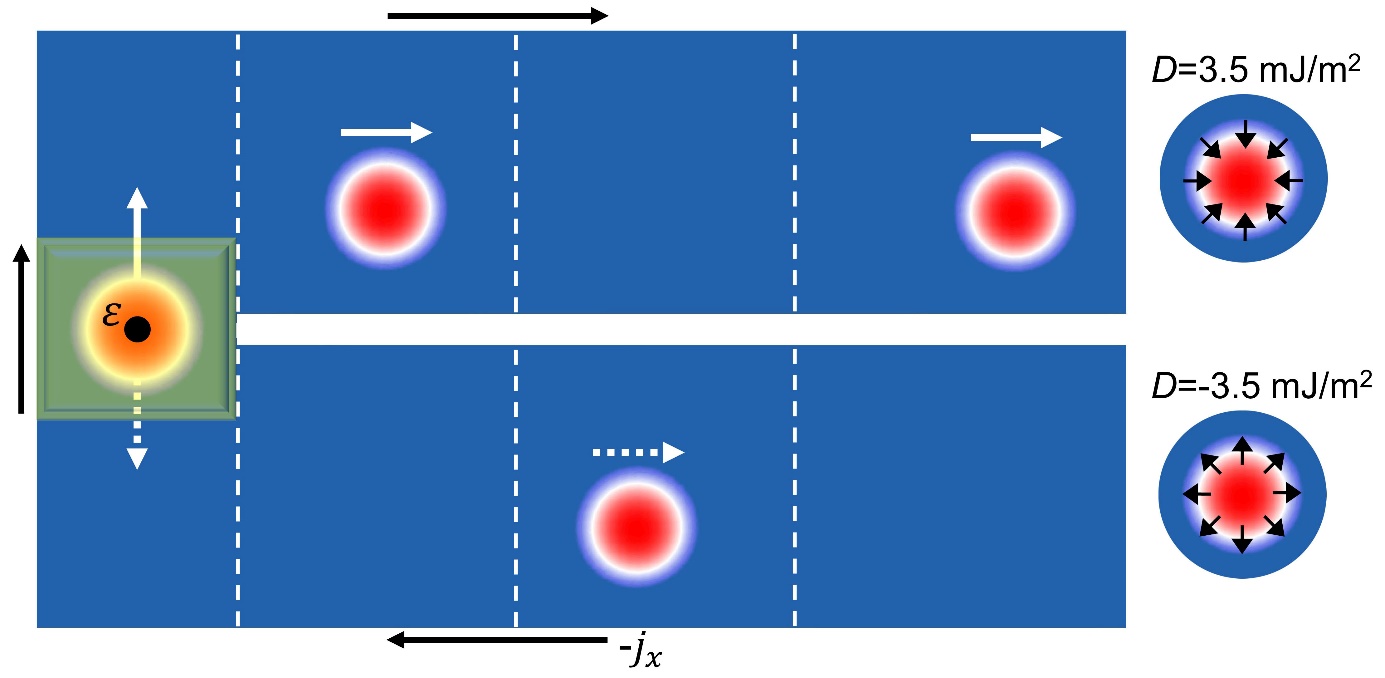


**Figure S7.** Complementary racetrack memory. This structure can be constructed by folding the nanotrack in Fig. 4. DMI chirality of the upper and lower channels is opposite, and the vertical electric field $\varepsilon$ can control the chirality of the skyrmions excited on the left. When the electric field is positive, the newly nucleated skyrmion will move to the upper channel, and vice versa.

**Motion of magnetic skyrmions with opposite chirality driven by a same current**

To investigate the effect of the skyrmion chirality switching on its motion mode, the Thiele’s equation derived from the LLG equation is employed:

$$\boldsymbol{G}\times\boldsymbol{v}-\alpha\mathcal{D}\cdot\boldsymbol{v}+\boldsymbol{F}_{\mathrm{SOT}}=0.$$

Here, the first term represents the Magnus force that gives rise to the Skyrmion Hall Effect (SkHE) [[1-5](#_ENREF_1)], gyrovector $\boldsymbol{G}=(M_{s}h/\gamma)4\pi Q\boldsymbol{z}$ with topological (or skyrmion) number $Q=1/(4\pi)\int\boldsymbol{m}\cdot(\partial_{x}\boldsymbol{m}\times\partial_{y}\boldsymbol{m})dxdy$, and $\boldsymbol{v}$ being the skyrmion velocity. $\alpha$ indicates the magnetic damping coefficient and $\mathcal{D}$ is the dissipative tensor. The force arising from spin-orbit torque (SOT) $F_{\mathrm{SOT}}$ can be identified as a volume integral for each component [[6](#_ENREF_6)]:

$$F_{x,SOT}=-\int d\mathcal{V}\boldsymbol{B}_{\mathrm{SOT}}\cdot\frac{\partial}{\partial x}\boldsymbol{M}=\tau_{\mathrm{DL}}\boldsymbol{\zeta\cdot}\int d\mathcal{V}\boldsymbol{m}\times\frac{\partial}{\partial x}\boldsymbol{m},$$

where the spin polarization along $\boldsymbol{\zeta}$ is parallel to the *y*-axis for current flowing along *x*. Because the x component of the force (along the current) is the the same as the part of the interface DMI that which involves the x gradient, the force $F_{x, SOT}$ from SOT will on a skyrmion depends on the its chirality and, polarity of skyrmion besides in addition to the spin polarization$\boldsymbol{\zeta}$. This means that when either the skyrmion helicity or topological charge is changed separately, the sign of the force $F_{x,SOT}$ will also be altered, and the skyrmion motion can be reversed without changing the direction of the current. But when the two are reversed at the same time, the force $F_{x,SOT}$ remains unchanged, only the SkHE will be reversed by the magnus force. Therefore, for the skyrmion driven by a transverse current, the skyrmion switching of the chirality will cause the direction of the force $F_{x,SOT}$to be reversed, i.e., giving the same effect as changing the current direction (the spin polarization $\boldsymbol{\zeta}$), thereby reversing the direction of skyrmion movement.

**References**

1 Jiang, W., Zhang, X. and Yu, G. *et al.* Direct observation of the skyrmion Hall effect. *Nat. Phys.* **13**, 162-169 (2017).

2 Litzius, K., Lemesh, I. and Krüger, B. *et al.* Skyrmion Hall effect revealed by direct time-resolved X-ray microscopy. *Nat. Phys.* **13**, 170-175 (2017).

3 Chen, G. Skyrmion Hall effect. *Nat. Phys.* **13**, 112-113 (2017).

4 Nagaosa, N.,and Tokura, Y. Topological properties and dynamics of magnetic skyrmions. *Nat. Nanotechnol.* **8**, 899-911 (2013).

5 Zang, J., Mostovoy, M. and Han, J. H. *et al.* Dynamics of Skyrmion Crystals in Metallic Thin Films. *Phys. Rev. Lett.* **107**, 136804 (2011).

6 Manchon, A., Železný, J. and Miron, I. M. *et al.* Current-induced spin-orbit torques in ferromagnetic and antiferromagnetic systems. *Rev. Mod. Phys.* **91**, 035004 (2019).
